# A dosimetric study to improve the quality of nodal radiotherapy in breast cancer

**DOI:** 10.1259/bjro.20210013

**Published:** 2021-07-05

**Authors:** Camarie Welgemoed, Simon Coughlan, Patti McNaught, Dorothy Gujral, Pippa Riddle

**Affiliations:** 1 Radiotherapy Department, Imperial College Healthcare NHS Trust, London, UK; 2 Department of Surgery and Cancer, Imperial College London, London, UK; 3 Radiotherapy Department, Royal Devon and Exeter NHS Foundation Trust, Exeter, UK

## Abstract

**Objectives:**

Field-based planning for regional nodal breast radiotherapy (RT) used to be standard practice. This study evaluated a field-based posterior axillary boost (PAB) and two forward-planned intensity-modulated RT (IMRT) techniques, aiming to replace the first.

**Methods:**

Supraclavicular and axillary nodes, humeral head, brachial plexus, thyroid, and oesophagus were retrospectively delineated on 12 CT scans. Three plans, prescribed to 40.05 Gy, were produced for each patient. Breast plans consisted of field-in-field IMRT tangential fields in all three techniques. Nodal plans consisted of a field-based PAB (anterior and posterior boost beam), and 2 forward-planned techniques: simple IMRT 1 (anterior and posterior beam with limited segments), and a more advanced IMRT 2 technique (anterior and fully modulated posterior beam).

**Results:**

The nodal V_90%_ was similar between IMRT 1: mean 99.5% (SD 1.0) and IMRT 2: 99.4% (SD 0.5). Both demonstrated significantly improved results (*p* = 0.0001 and 0.005, respectively) compared to the field-based PAB technique. IMRT 2 lung V_12Gy_ and humeral head V_10Gy_ were significantly lower (*p* = 0.002, 0.0001, respectively) than the field-based PAB technique. IMRT 1 exhibited significantly lower brachial plexus D_max_ and humeral head V_5_, _10_, and _15Gy_ doses (*p* = 0.007, 0.013, 0.007 and 0.007, respectively) compared to the field-based PAB technique. The oesophagus and thyroid dose difference between methods was insignificant.

**Conclusions:**

Both IMRT techniques achieved the dose coverage requirements and reduced normal tissue exposure, decreasing the risk of radiation side effects. Despite the increased cost of IMRT, compared to non-IMRT techniques ^1^, both IMRT techniques are suitable for supraclavicular and axillary nodal RT.

**Advances in knowledge:**

Forward-planned IMRT already resulted in significant dose reduction to organs at risk and improved planning target volume coverage.^1^ This new, simplified forward-planned IMRT one technique has not been published in this context and is easy to implement in routine clinical practice.

## Introduction

Radiotherapy (RT) for early-stage breast cancer reduces the recurrence risk and improves overall survival. In patients with a positive sentinel node biopsy (SNB), the Early Breast Cancer Trials Collaborative Group reported a significant reduction in mortality with adjuvant loco-regional RT, regardless of the number of positive lymph nodes (LNs) or systemic treatments.^2^ The AMAROS trial compared axillary LN dissection (ALND) to regional nodal irradiation (RNI) in post-menopausal, SNB-positive patients.^3^ They concluded RNI was equivalent to ALND in efficacy and resulted in less lymph-oedema but increased rates of shoulder stiffness. The trial mandated contouring of LNs and 3D planned RT to include LNs – neither of which is currently employed by all UK departments.

Traditional field-based techniques: anterior field, PAB and anterior- and posterior axillary boost employed bony landmarks to position fields and were widely used. However, previous studies have demonstrated marked variation in the depth and position of LNs, due to the range of body habitus, depth of subcutaneous adipose tissue, and arm position.^4^ The variation in LN positions suggested these techniques to be suboptimal^5–8^ and subsequently, they were replaced with 3D conformal RT (3DCRT) techniques. 3DCRT was the standard until the introduction of IMRT, improving nodal target volume coverage and reducing high-dose areas. Despite the increased cost of IMRT^1^ and requiring advanced delineation and planning skills, IMRT offers improved LN dose coverage while minimising dose to organs at risk (OAR).^9^

This study compared LN planning target volume (PTV) dose conformity, homogeneity and OAR exposure of a field-based posterior axillary boost (PAB) and two forward-planned IMRT techniques, intending to replace the current field-based PAB method.

## Methods and materials

### Patient selection, positioning, and CT scanning

CT scans of 12 consecutive patients referred for adjuvant breast/chest wall irradiation were selected for the study. Patients were scanned in a supine position on a breast board with arms raised and head straight. 3 mm slices from mid-neck to 50 mm inferior to the breast were performed on a wide bore Philips AcQSim CT Scanner (Philips Medical Systems, Guildford, UK). Retrospective field-based PAB and two forward-planned IMRT plans were generated, and evaluation based on ICRU 62 requirements.^10^ The study received local information governance and institutional audit committee approval.

### Delineation

Two experienced breast specialists retrospectively delineated supraclavicular and axillary nodes, humeral head, brachial plexus (BP), thyroid and oesophagus. Delineation of nodal clinical target volumes (CTVs) conformed to the European Society for Radiotherapy and Oncology (ESTRO) consensus guidelines.^11^

### Planning

Tangential fields were planned first with a field-in-field, forward-planned IMRT technique. The posterior tangential field edges were non-divergent to minimise in-field lung and heart. For each tangential field, 80–85% of the dose was delivered by an open field, and the rest of the dose, typically by 3–5 segments per field. After that, nodal fields were matched to the tangential fields utilising a mono-isocentric beam arrangement. Three nodal plans: a field-based PAB, simple forward-planned IMRT 1, and a more advanced forward-planned IMRT 2 technique were produced for each patient.

In all three plans, the LN CTV was expanded by 5 mm to create a PTV and modified medially, excluding the trachea.^12^ Previous studies have shown that recurrences occur within the field, and larger volumes could cause increased toxicity^13^; therefore, every effort was made to ensure the treatment field encompassing the PTV was not more extensive than traditional nodal fields.

**Figure 1.**
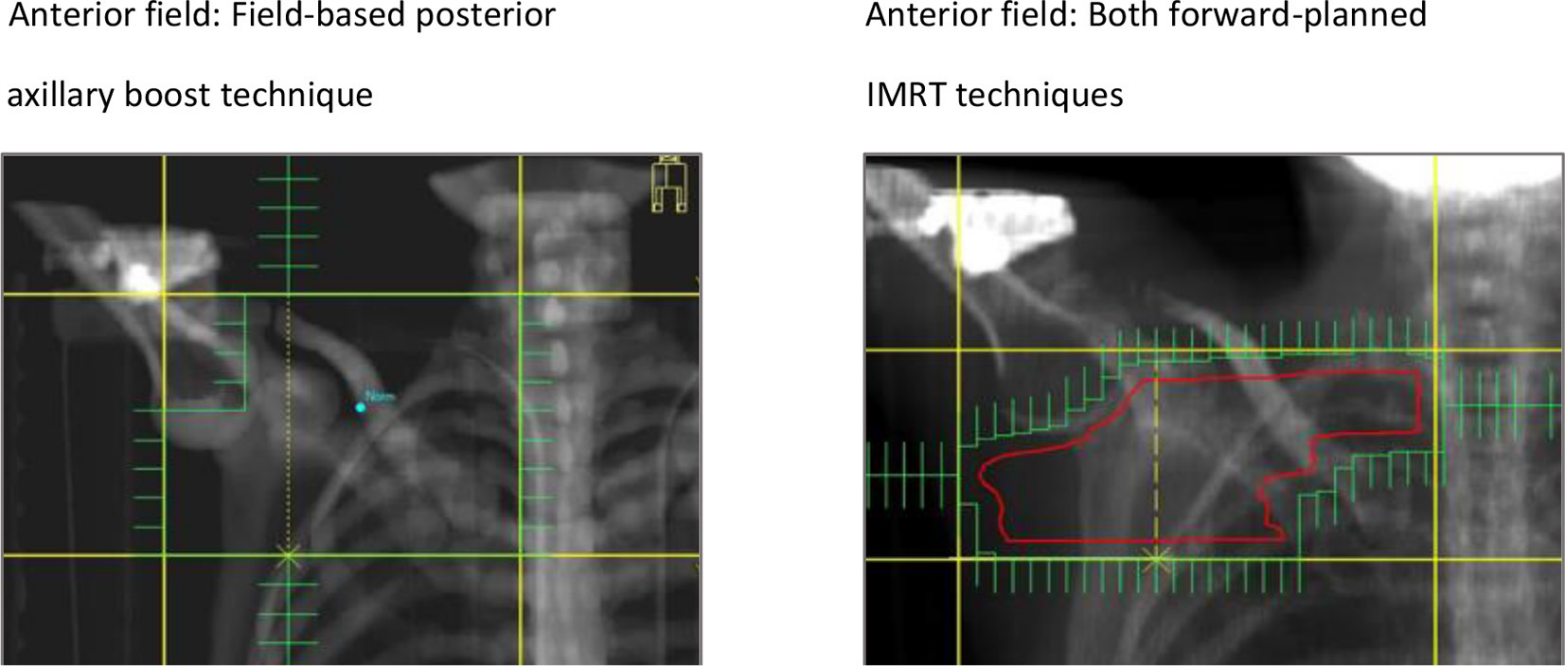
Digitally reconstructed radiographs displaying the anterior fields and shielding for the field-based posterior axillary boost and IMRT techniques

**Figure 2.**
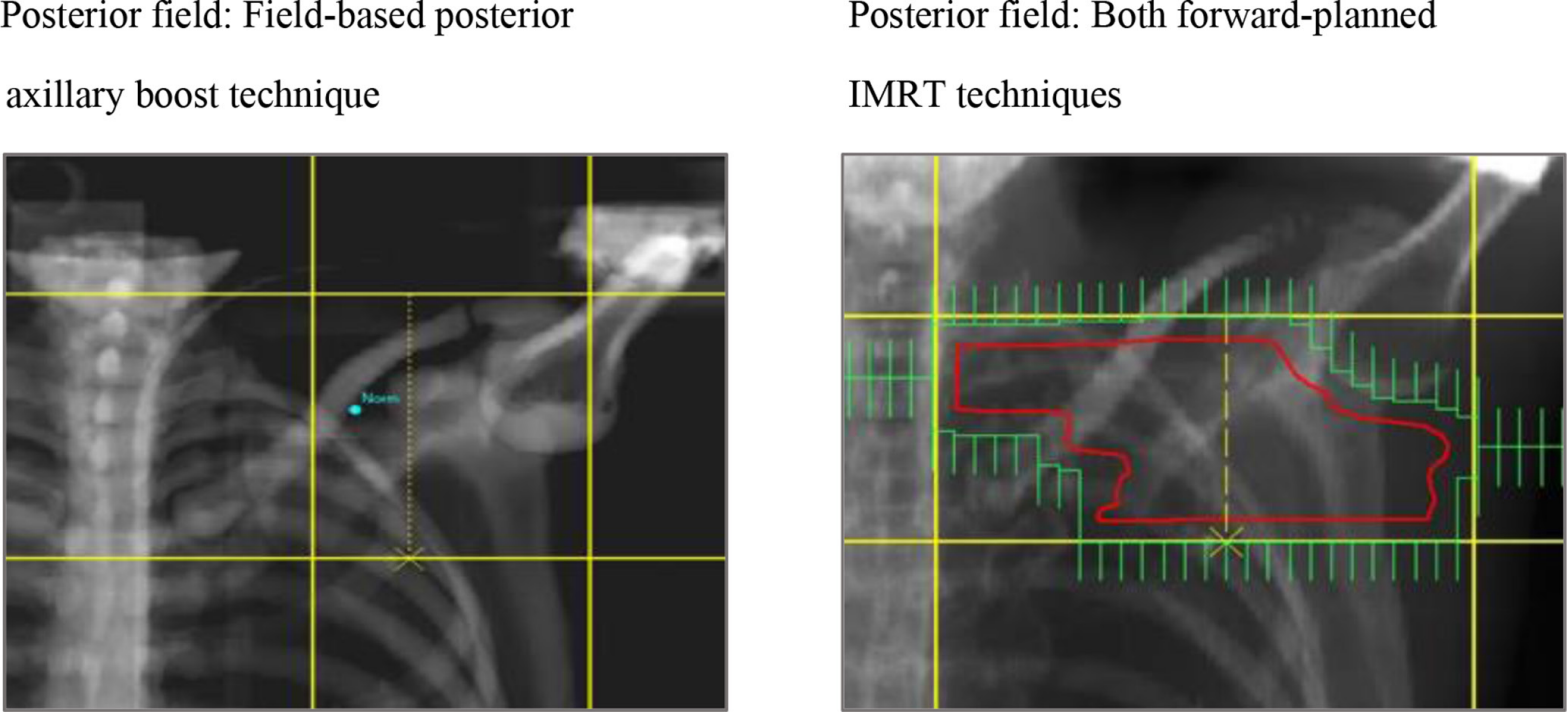
Digitally reconstructed radiographs displaying the posterior fields and shielding for the field-based posterior axillary boost and IMRT techniques

In the field-based PAB technique, the single anterior oblique field border was defined laterally by the humeral head, superiorly by the fourth and fifth cervical vertebral space, and medially by the trachea. A PAB field encompassed the lateral half of the anterior field. The humeral head was shielded with multi-leaf collimation (MLC) on both anterior and posterior fields. In both forward-planned IMRT techniques, MLC conformed to the PTV + 0.5 cm (Figures 1 and 2).

**Figure 3.**
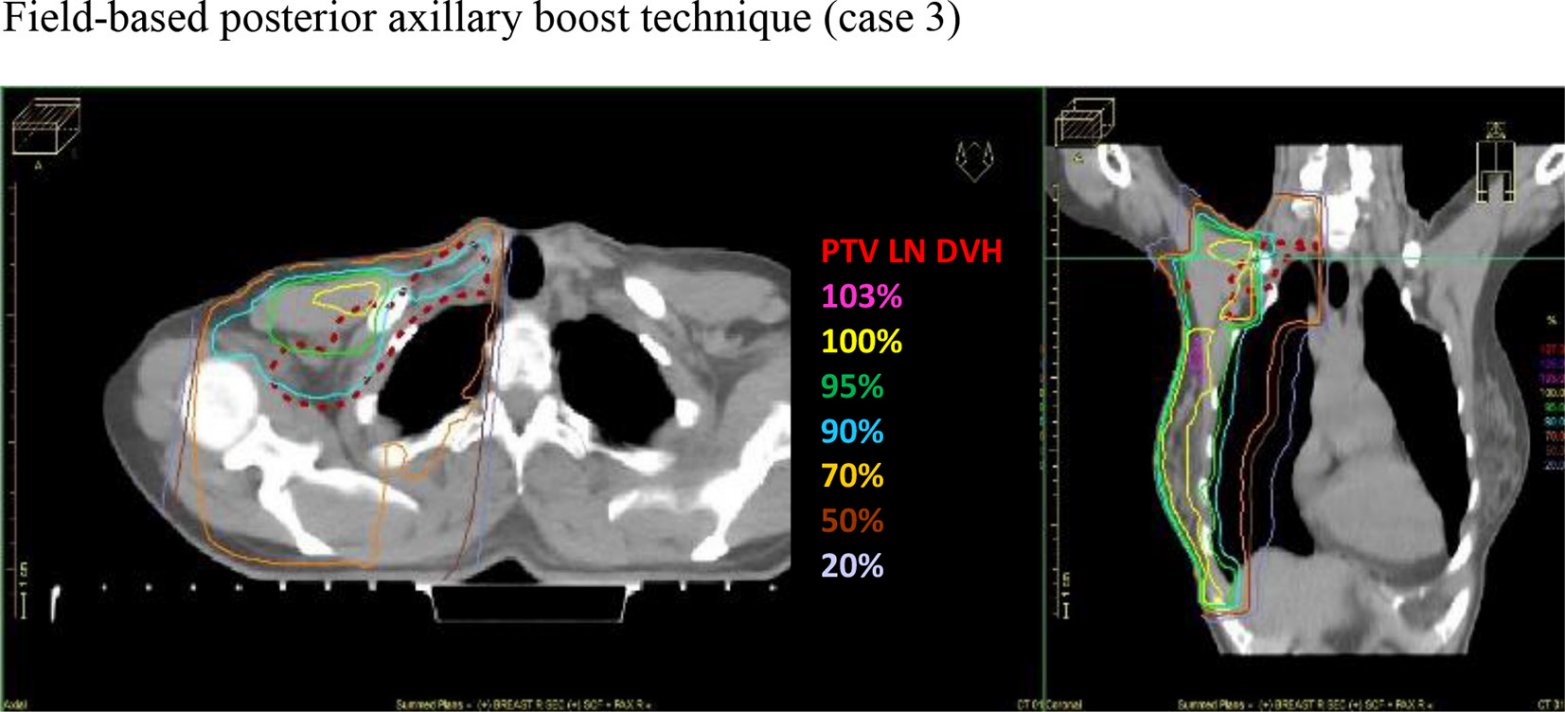
Axial and coronal CT slice views, demonstrating the iso-dose distributions for the field-based posterior axillary boost technique

**Figure 4.**
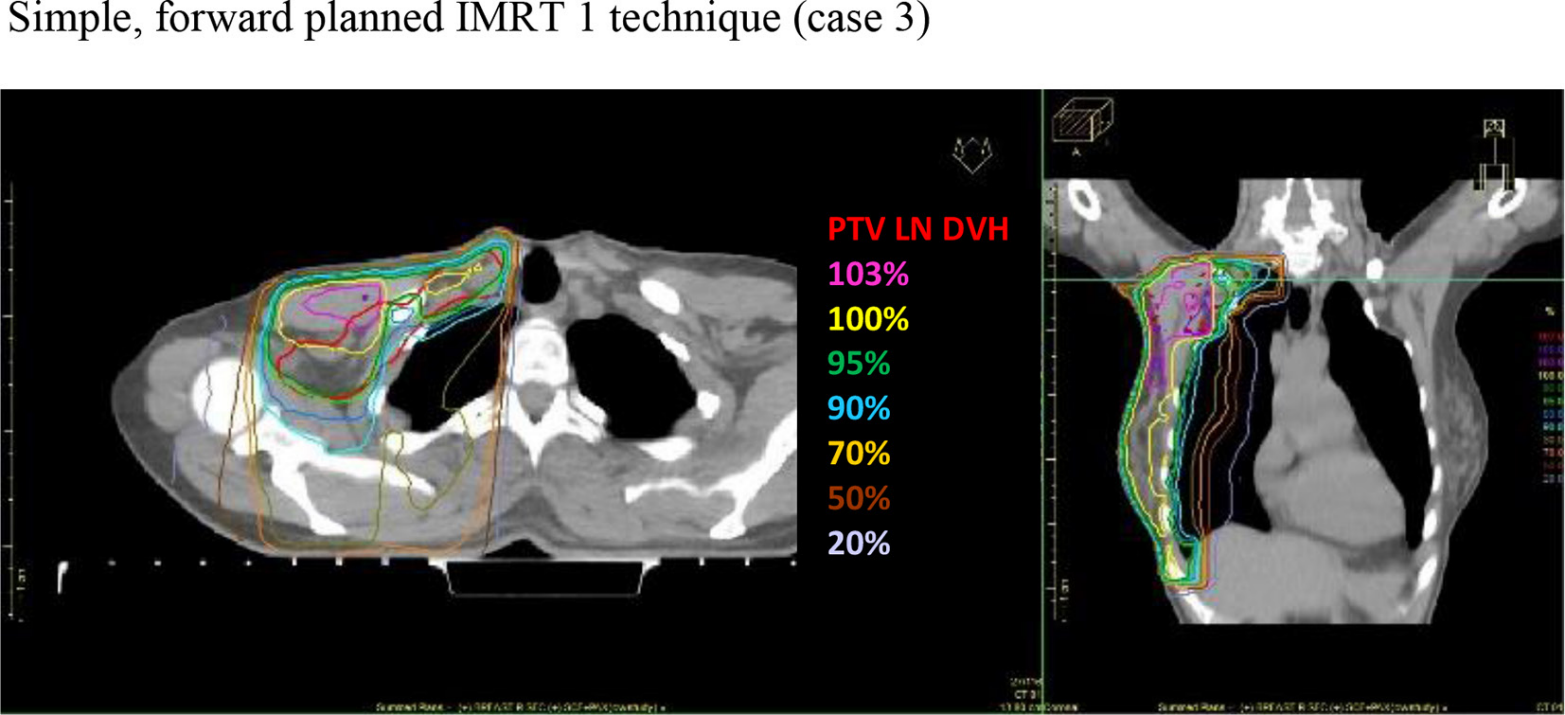
Axial and coronal CT slice views, demonstrating the iso-dose distributions for the IMRT 1 technique

**Figure 5.**
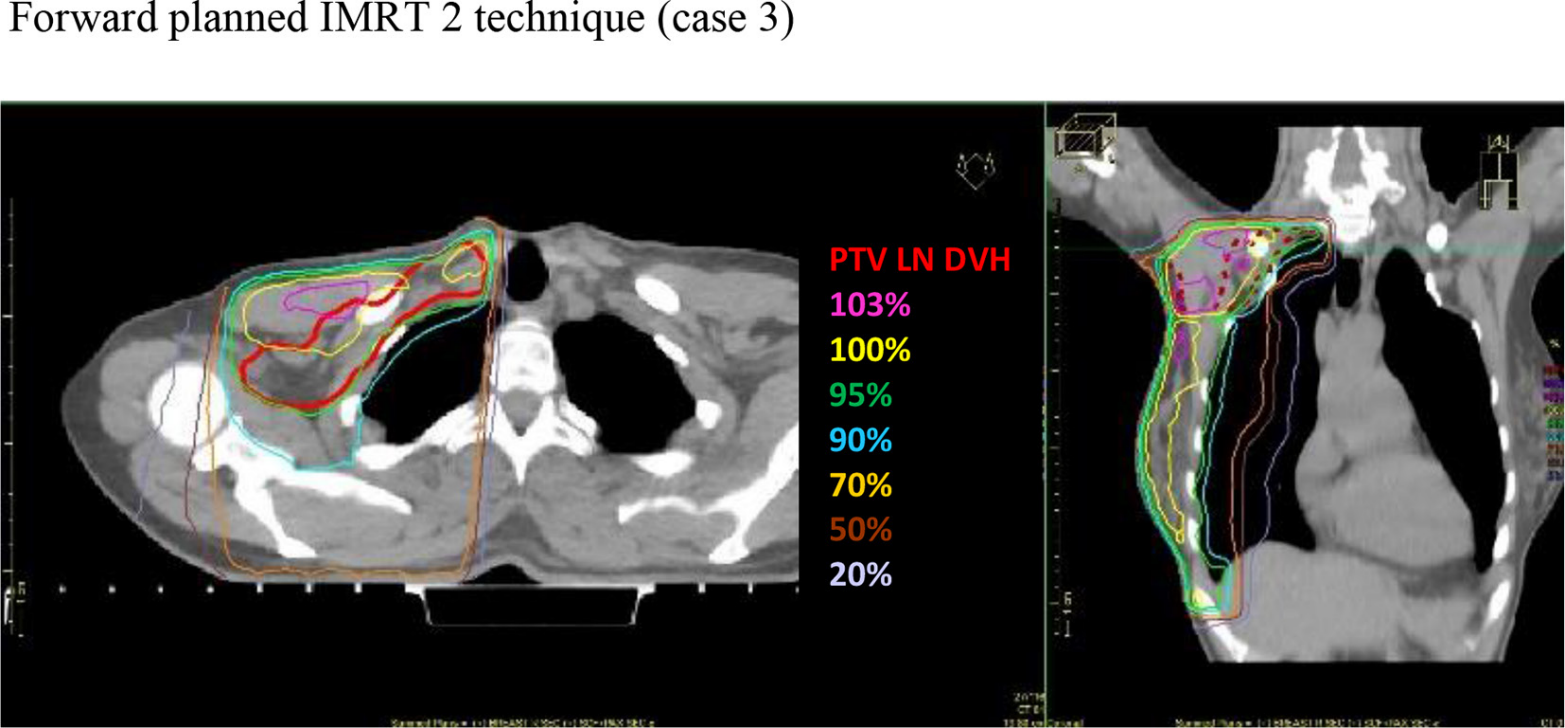
Axial and coronal CT slice views, demonstrating the iso-dose distributions for the IMRT 2 nodal technique

Treatment plans were generated with commercial software (Nucletron, OTP v.4.1). Type B (collapsed cone) dose calculations took lung density into account. A combination of dynamic wedges (opposing fields), field-in-field modulation (IMRT plans), and six or 10 MV photon energies (depending on hot spots and nodal volume depth) were applied to create homogeneous dose distributions. A minimum of five monitor units was delivered per segment. In both forward-planned IMRT methods, nodal fields weighted approximately 80:20, anterior: posterior (Figures 3–5).

A dose of 40.05 Gy in 15 fractions over three weeks was prescribed to the ICRU recommended reference point.^10^ Dose-volume histograms (DVH) calculated and assessed the dosimetry of composite breast/chest wall and nodal fields. The nodal prescribed dose in the field-based PAB technique was modified to ensure the BP tolerance dose was not exceeded.

The following planning and dose objectives were applied:

- LN PTV: V90% ≥ 90% (LN PTV volume receiving 90% of the dose is greater than 90%).
- Breast/chest wall: V95% ≥ 95% (volume receiving 95% of the dose is greater than 95%).
- V107% ≤ 1 cc
- LN and breast PTV maximum dose: ≤ 110% prescribed dose.
- LN PTV DVH: V_107%_ ≤ 2%
- BP maximum point: ≤ 110% of the prescribed dose.
- Ipsilateral lung: V_12Gy_ ≤ 25% (similar to the V_30%_ < 17%, POSNOC trial.^14^

For the nodal IMRT plans, anterior and posterior beams were angled to encompass the LN PTV (supraclavicular and axillary nodes) with a margin for beam penumbra. The posterior beam in the simple IMRT 1 technique comprised of 1–2 segments. In the more advanced IMRT 2 technique, LN PTV under dosed areas were iteratively defined as pseudo targets to guide segment design. The modulation of the posterior field was optimised to compensate accordingly. The resultant 4–7 segments were merged into a single step-and-shoot IMRT field for delivery, preventing an increase in treatment delivery time (Figures 3–5). To demonstrate the dosimetric implications of the junction between tangential and nodal fields, we have created a “junction structure” (6 mm slice of junction PTV) and recorded minimum and maximum doses.

### Analysis

Plan evaluation parameters were calculated for each structure, and 26 DVHs were generated.

We analysed the mean V_95%_ (volume receiving 95% of the dose) for ipsilateral breast PTV dose coverage, V_40.05Gy_ (volume receiving 40.05 Gy), and D_mean._

For dose coverage of the LN PTV above the junction and the total LN PTV, mean V_90%_ (volume receiving 90% of the dose) was analysed.

Hotspots were represented by breast D_2cc max_ (absolute volume in cc), V_107%_ within and outside the LN PTVs, and LN D_max_ to determine dose uniformity.

OAR data included: BP D_max_, ipsilateral lung V_5, 10, 12, 20, 30 Gy_ (volume receiving 5, 10, 12, 20, and 30 Gy, respectively), oesophagus D_mean_, and thyroid V_30Gy_, D_mean_, D_max_ and D_2cc max_. Humeral head V_5Gy_, V_10Gy_ and V_15Gy_ were selected for plan evaluation in the absence of published dose constraints.

Statistical analysis was performed with IBM SPSS statistics software, v.27. Means, standard deviations (SD) and 95% confidence limits (95% CI) were calculated for all parameters. The three techniques’ dose parameter means were compared with the Friedman’s test, a non-parametric alternative to the ANOVA test, to determine the statistical significance of differences. In the event of null hypothesis rejection (*p* = ≤0.05), pairwise comparisons were performed to determine the location of significant differences between techniques. The significance level was set at a *p*-value of <0.05 and adjusted by the Bonferroni correction to avoid type I errors when making multiple statistical tests.

## Results

### Breast/thoracic wall PTV

Overall, V_95%_ was achieved. D_mean_ for the field-based PAB technique was mean 40.3 Gy (SD ±0.4, CI: 40.1–40.6), IMRT 1: 40.4 Gy (SD ±0.4, CI: 40.1–40.6) and IMRT 2: 40.2 Gy (SD ±0.4, CI: 40–40.5). D_2cc_ were below the maximum dose constraint of 110% (44.05Gy), indicating a homogeneous dose distribution. (Table 1)

**Table 1.** Dose parameters for planning techniques: Field-based posterior axillary boost, IMRT one and IMRT 2. (*n* = 12)

	Field-based PAB	Simple IMRT 1	IMRT 2	Field-based PAB compared to IMRT 1	IMRT two compared to field-based PAB	IMRT two compared to IMRT 1
	Mean (SD)	Mean (SD)	Mean (SD)	*p*-value	*p*-value	*p*-value
Ipsilateral breast PTV						
V40.05Gy	62.4 (14.5)	62.9 (14.8)	58.6 (14.3)^*a*^		0.002	0.003
V 95%	95.6 (4.1)	95.4 (3.9)	95.1 (4.4)^*a*^		0.043	
Dmean	40.3 (0.4)	40.4 (0.4)	40.2 (0.4)^*a*^		0.013	0.009
D2cc max (Gy)	41.9 (0.3)	41.9 (0.4)	41.8 (0.5)^*a*^		0.043	
Lymph node PTV (SCF and axilla)						
V90% (above junction)	56.7 (24.7)^*a*^^a^	99.5 (1.0)	99.4 (0.5)	0.0001	0.005	
V90% Total LN PTV (≥90%)	67.0 (15.7)^*a*^	94.5 (5.3)	94.4 (4.6)	0.0001	0.013	
V107% (≤1 cc)	1.2 (3.0)	0.6 (0.9)	0.0 (0)			
Maximum doses						
V107% (outside PTVs ≤ 1 cc)	0.1 (0.2)	0.0 (0.1)	0 (0.1)			
Dmax (≤110% PS dose)	111.6 (6.0)	111.9 (4.4)	106.9 (1.4)^*a*^			0.002
Brachial Plexus						
Dmax (≤110% PS dose)	99.0 (7.9)^*a*^	103.0 (1.9)	98.8 (1.9)^*a*^	0.007		0.007
Ipsilateral lung (V12Gy ≤ 25%)						
V5Gy	44.7 (5.9)	42.2 (5.9)^*a*^	41.5 (5.7)^*a*^		0.001	0.043
V10Gy	55.3 (19.0)	31.1 (5.7)^*a*^	30.7 (5.7)^*a*^	0.005	0.0001	
V12GY	31.9 (6.3)	29.0 (6.2)	28.4 (6.0)^*a*^		0.002	
V20Gy	26.4 (5.5)	23.6 (5.2)	22.8 (5.2)^*a*^		0.0001	
V30Gy	12.0 (4.7)^*a*^	16.4 (5.0)	14.4 (4.6)^*a*^	0.0001		0.024
Humeral head						
V5Gy	74.0 (15.2)	27.1 (10.8)^*a*^	22.4 (10.1)^*a*^	0.013	0.0001	
V10Gy	55.3 (19.0)	14.4 (8.7)^*a*^	10.7 (7.9)^*a*^	0.007	0.0001	
V15Gy	37.7 (7.9)	10.0 (7.5)^*a*^	7.6 (6.9)^*a*^	0.007	0.0001	
Oesophagus						
Dmean	1.2 (0.3)	2.0 (2.0)	1.0 (0.4)			
Dmax	10.1 (10.8)	16.2 (15.2)	11.4 (11.4)			
Thyroid						
V30Gy	17.6 (14.3)	14.3 (13.4)	13.3 (12.2)			
Dmean	10.3 (6.8)	8.4 (4.7)	7.9 (4.8)			
Dmax	33.7 (20.6)^*a*^	34.8 (10.0)	35.7 (7.6)	0.024		
D2cc max (Gy)	33.5 (3.5)	35.2 (6.0)	31.8 (10.9)			
Junction (3 mm superior and 3 mm inferior of iso-centre)						
Dmean	36.6 (3.3)^*a*^	38.4 (2.4)	36.8 (1.8)^*a*^	0.005		0.003
Dmax	41.9 (2.4)^*a*^	42.6 (1.3)	40.8 (1.3)^*a*^	0.043		0.007

D_2cc max_, Maximum dose to 2cc area; D_max_, Maximum dose; D_mean_, Mean dose; IMRT, Intensity-modulated radiotherapy; PAB, Posterior axillary boost; PTV, Planning target volume; SCF, Supra clavicular fossa; SD, Standard deviation; V_90%_, Volume receiving 90% of the dose; V_95%_, Volume receiving 95% of the dose; V_5Gy_, Volume receiving 5Gy; V_10Gy_, Volume receiving 10Gy; V_15Gy_, Volume receiving 15Gy; V_30Gy_, Volume receiving 30Gy; V_40.05Gy_, Volume receiving 40.05Gy.

a Statistically significant values < 0.05.

### Lymph node PTV

**Figure 6.**
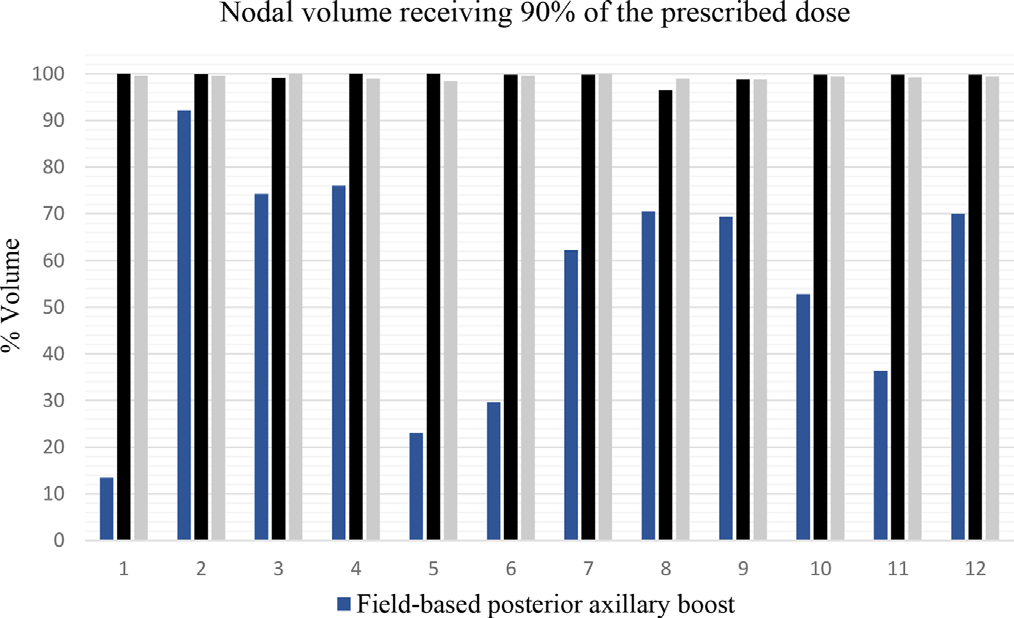
This graph displays the nodal volumes receiving 90% of the dose for the 12 field-based posterior axillary boost plans

Figures 3–5 demonstrate the isodose distributions for the three techniques. When comparing V_90%_ (above the junction) between field-based PAB: mean 56.7% (SD ±24.7, CI: 41.0–72.4) and IMRT 1: mean 99.5% (SD ±1.0, CI: 98.8–100.1), and field-based PAB and IMRT 2: mean 99.4% (SD ±0.5, CI: 99.1–99.6), the difference was statistically significant, *p* = 0.0001 and *p* = 0.005, respectively, demonstrating improved dose conformity in both IMRT techniques. V_90%_, field-based PAB technique, was particularly low in patients 1, 5, 6 and 11; 13.5%, 23.1%, 29.6 and 36.4%, respectively. (Figure 6) V_80%_, field-based PAB technique was 65.4, 77.7, 82.9 and 80.5%, respectively. V_107%_ ≤ 1 cc was achieved in both IMRT techniques with no significant differences between the three techniques. (Table 1)

The mean V_107%_ outside the PTVs were <1 cc in all three techniques.

PTV D_max_ in IMRT 2: mean 106.9% (SD ±1.4, CI: 106.1–107.8) was statistically significantly lower (*p* = 0.002) than simple IMRT 1: 111.9% (SD ±4.4, CI: 109.2–114.7) and lower than field-based PAB: 111.6% (SD ±6.0, CI: 107.8–115.3), indicating a more homogeneous dose distribution in IMRT 2 (Table 1)

### Dose in the junction between the nodal and tangential fields

For the field-based PAB technique, D_max_: mean 41.9 Gy, (SD ±2.4, CI: 40.4–43.4) was significantly lower (*p* = 0.043) compared to IMRT 1: 42.6 Gy (SD ±1.3, CI: 41.6–43.5). D_max_ in IMRT 2: mean 40.8 Gy (SD ±1.3, CI: 40.0–41.6) was significantly lower (*p* = 0.007) compared to IMRT 1.

D_mean_ in IMRT 2: mean 36.8 Gy (SD ±1.8, CI: 35.7–37.9) compared to IMRT 1: 38.4 Gy (SD ±2.4, CI: 36.9–39.9) was significantly lower (*p* = 0.003). (Table 1)

### Critical structures

#### Brachial plexus

D_max_ was < 110% in all three techniques. This, however, was achieved in the field-based PAB technique by prioritising BP sparing over LN PTV dose coverage, reducing the nodal prescribed dose. Pairwise comparisons of Dmax between IMRT 2: mean 98.8% (SD ±1.90, CI: 97.6–100.0) and IMRT 1: 103.0% (SD ±1.9, CI: 101.8–104.2) was significantly lower (*p* = 0.007) for IMRT 2. (Table 1)

#### Ipsilateral lung

In the field-based PAB technique V_20Gy_: mean 26.4 Gy (SD ±5.5, CI: 22.9–29.8), V_12_: 31.9 Gy (SD ±6.3, CI: 27.4–36.4), V_10Gy_: 55.3 Gy (SD ±19, CI: 43.2–67.3) and V_5Gy_: 44.7 Gy (SD ±5.9, CI: 41.0–48.5) were significantly higher (*p* = 0.0001, 0.002, 0.0001 and 0.001, respectively) when compared to IMRT 2: V_20Gy_: mean 22.8 Gy (SD ±5.2, CI: 19.5–26.1), V_12Gy_: 28.4 Gy (SD ±6.0, CI: 24.1–32.7), V_10Gy_: 30.7 Gy (SD ±5.7, CI: 27.0–34.3) and V_5Gy_: 41.5 Gy (SD ±5.7, CI: 37.9–45.1).

V_30Gy,_ IMRT 2: mean 14.4 Gy (SD ±4.6, CI: 11.5–17.3) compared to V_30Gy,_ IMRT 1: 16.4 Gy (SD ±5.0, CI: 13.2–19.5) was significantly lower (*p* = 0.024) in IMRT 1. V_30Gy_, field-based PAB: mean 12.0 Gy (SD ±4.7, CI: 9.0–14.9), compared to V_30Gy_, IMRT 1: 16.4 Gy (SD ±5.0, CI: 13.2–19.5) was significantly lower (*p* = 0.0001) in the field-based PAB technique. (Table 1)

#### Humeral head

IMRT 2, V_5Gy_: mean 22.4 Gy (SD ±10.1, CI: 16.0–28.8), V_10Gy_: 10.7 Gy (SD ±7.9, CI: 5.7–15.7) and V_15Gy_: 7.6 Gy (SD ±6.9, CI: 3.2–12.0) compared to field-based PAB: V_5Gy_: 74.0 Gy (SD ±15.2, CI: 64.3–83.6), V_10Gy_: 55.3 Gy (SD ±19.0, CI: 43.2–67.3) and V_15Gy_: 37.7 Gy (SD ±7.9, CI: 32.7–42.7), confirmed significantly lower doses in IMRT 2 (V_5Gy,_ V_10Gy,_ and V_15Gy_ *p* = 0.0001). IMRT 1, V_5Gy_: 27.1 Gy (SD ±10.8, CI: 20.3–34.0), V_10Gy_: 14.4 Gy (SD ±8.7, CI: 8.9–19.9) and V_15Gy_: 10.0 Gy (SD ±7.5, CI: 5.2–14.7) also confirmed significantly lower doses (*p* = 0.013, 0.007 and 0.007, respectively) when compared to the field-based PAB technique. (Table 1)

#### Oesophagus

D_mean_ was ≤ 2 Gy and similar for all three techniques. The difference in D_max_ between field-based PAB: mean 10.1 Gy (SD ±10.8, CI: 3.2–16.9), IMRT 1: 16.2 Gy (SD ±15.2, CI: 6.6–25.9) and IMRT 2: 11.4 Gy (SD ±11.4, CI: 4.1–18.6), was not significant.

#### Thyroid

Field-based PAB, V_30Gy_: mean 17.6 Gy (SD ±14.3, CI: 8.6–26.7) and D_mean_: mean 10.3 Gy (SD ±6.8, CI: 6.0–14.6) was higher but not significant compared to the IMRT techniques. IMRT 1, V_30Gy_ was: mean 14.3 Gy (SD ±13.4, CI: 8.6–26.7) and D_mean_: 8.4 (SD ±4.7, CI: 5.4–11.3) whereas IMRT 2, V_30Gy_ was: mean 13.3 Gy (SD ±12.2, CI: 5.6–21.1) and D_mean_: 7.9 (SD ±4.8, CI: 4.9–10.9). The difference in D_2cc max_ between field-based PAB: mean 33.5 Gy (SD ±3.5, CI: 31.2–35.7), IMRT 1: 35.2 Gy (±6, CI: 31.4–39.1) and IMRT 2: 31.8 Gy (±10.9, CI: 24.8–36.7), were insignificant. D_max_ was significantly higher (*p* = 0.024) in IMRT 1: 34.8 (±10.0, CI: 28.4–41.2) compared to field-based PAB: mean 33.7 Gy (±20.6, CI: 20.6–46.7). However, D_max,_ compared to the other techniques was the highest in IMRT 2: mean 35.7 Gy (SD ±7.6, CI: 30.9–40.6), but not significant.

## Discussion

We explored a satisfactory compromise of two forward-planned IMRT techniques to improve dose conformance, homogeneity and OAR dose, compared to a field-based PAB method. The forward-planned IMRT modulations were relatively simple, averaging 1–2 segments in IMRT 1 and 4–7 segments in IMRT 2, making them more robust for treatment delivery and virtually invisible at the treatment end. The integral dose in forward-planned IMRT, unlike inverse planned IMRT, remains low and is important because of the possible correlation between increased dose to normal tissue and secondary malignancies.^15,16^

It was not within the scope of this study to compare tangential field planning techniques. The dose objectives for breast/chest wall RT have been achieved with forward-planned, field-in field IMRT. Inverse-planned, hybrid or volumetric modulated arc therapy techniques may be more suitable for medially located tumour beds or internal mammary nodes.

We did not explore multifield conformal RT because IMRT techniques achieve similar/improved dose distributions without requiring 3–5 beams, which would invariably irradiate more normal tissue. Furthermore, delivering those beams requires additional treatment time with all the associated setup issues.

It is, however, essential to acknowledge that setup errors during breast and RNI are not negligible and are independent of the chosen technique. Pre-treatment verification of the isocentre should be considered, including kV or MV planar imaging, CBCT, or surface-guided RT.

Both IMRT techniques resulted in improved nodal dose conformance when compared to the field-based PAB technique. Poor dose conformance with the field-based PAB technique in patients 1, 5, 6 and 11 (Figure 6) resulted from more considerable dose reductions to achieve BP dose constraints. Furthermore, a significant part of the nodal PTV was not covered by the lateral field border in patient 1. Dose reduction to spare normal tissue, not nodal depth or patient separation, was the common cause of poor dose conformance in these four patients.

Regarding dose homogeneity and when considering V107% (within the LN PTV), LN D_max_, and the maximum dose in the junction between the tangential and nodal plans, IMRT 2 performed better than the IMRT 1 technique. Both the LN D_max_ and junction D_max_ were statistically significantly lower in IMRT 2 compared to IMRT 1. The lower doses were achieved by reducing medial hotspots with lateral segments to the posterior field.

At the time of this study, no published thyroid, oesophagus, and humeral head dose constraints were available. When evaluating OAR doses, the brachial plexus D_max_, ipsilateral lung V_5_ and V_30Gy_ were the only dose parameters that were statistically significantly lower in IMRT 2 than IMRT 1. The lung V_12Gy_ constraint has not been achieved in either of the techniques.

High-dose irradiation is associated with hypothyroidism and Graves’ disease, but no studies have reported a significant increase in hypothyroidism due to moderate-to low-dose irradiation.^17^ Our results confirmed a lower D_mean_ for both nodal IMRT techniques, 7.9 Gy (±4.8) and 8.4_ _Gy (±4.7). Higher thyroid D_mean_ doses (13.6 Gy ± 2.9) have been reported when combining VMAT breast plans with 3D nodal techniques.^18^ The lower D_mean_ can also be attributed to the difference in RTOG and ESTRO contouring guidelines. With the ESTRO guidelines, the distance between the thyroid and nodal CTV is larger, and consequently, thyroid exposure will be lower. Considering the development of volumetric techniques, thyroid dose constraints are an area of future research that may impact RT planning for individual patients. There is insufficient clinical data regarding doses to thyroid, oesophagus and humeral head, so the best practice would be to achieve the lowest possible doses for these OAR.

When comparing simple IMRT 1 to IMRT 2, there were no significant differences in the LN PTV coverage; however, IMRT 1 compares slightly better to the field-based PAB technique than IMRT 2. Despite a higher BP dose in IMRT 1, most dose constraints have been achieved. The mean D_max_ for IMRT 1 was 111.9%, exceeding 110% by less than 1 Gy. The only significant difference between the two techniques was V_5Gy_ and V_30Gy,_ which are not routinely used in the clinic. Outcomes from both IMRT techniques demonstrate target volume dose conformity, homogeneity and OAR dose benefits, supporting the replacement of the field-based PAB technique. IMRT 1 is a simplified technique consisting of fewer segments and reduced planning time. Based on dose information from this small sample size of 12 cases, IMRT 1 is a suitable choice for treating the breast, SC and AX nodes in a busy RT department.

## Conclusion

Both forward-planned IMRT techniques were an improvement on the field-based PAB technique as they enabled the delivery of the prescribed dose to a designated PTV volume. This study confirmed the feasibility of a simplified IMRT 1 technique compared to a more advanced IMRT 2 technique. Although IMRT planning is more costly,^1^ enhanced dose homogeneity and reduced lung, humeral head, and brachial plexus doses make it the RNI technique of choice. Inverse and rotational IMRT techniques, with resultant increased low dose areas, should be reserved for internal mammary nodal irradiation or anatomically challenging cases. Ultimately, technique choices depend on equipment and skill mix.
